# Morphological Hallmarks of Classical Fabry Disease: An Ultrastructural Study in a Large Spanish Family

**DOI:** 10.3390/jcm12175689

**Published:** 2023-08-31

**Authors:** Beatriz San Millán-Tejado, Carmen Navarro, Julián Fernández-Martín, Alberto Rivera, Irene Viéitez, Susana Teijeira, Saida Ortolano

**Affiliations:** 1 Rare Disease and Pediatric Medicine Group, Galicia Sur Health Research Institute (IIS Galicia Sur), Servizo Galego de Saúde-Universidade de Vigo (SERGAS-UVIGO), 36312 Vigo, Spain; jorge.julian.fernandez.martin@sergas.es (J.F.-M.); irene.vieitez@iisgaliciasur.es (I.V.); susana.teijeira.bautista@sergas.es (S.T.); 2 Department of Pathology, Hospital Alvaro Cunqueiro, Servizo Galego de Saúde (SERGAS), 36213 Vigo, Spain; cnavfer@sergas.es; 3 Molecular Medicine PhD Program, University of Santiago de Compostela, 15782 Santiago de Compostela, Spain; 4 Department of Internal Medicine, Hospital Alvaro Cunqueiro, Servizo Galego de Saúde (SERGAS), 36213 Vigo, Spain; alberto.rivera.gallego@sergas.es; 5 Systemic Autoimmune Disease and Thrombose Group, Galicia Sur Health Research Institute (IIS Galicia Sur), Servizo Galego de Saúde-Universidade de Vigo (SERGAS-UVIGO), 36312 Vigo, Spain

**Keywords:** Fabry disease, Gb3, lysosome, biopsy, electron microscopy

## Abstract

Fabry disease (FD) is a rare lysosomal disorder caused by α-galactosidase A deficiency, and it leads to the systemic deposition of globotriasylceramide. Demonstrations of the storage material in biopsies support this diagnosis. We report a histological and ultrastructural study of biopsies that were performed on 11 individuals from a family with the variant p.Gln279Arg in *GLA*, which is associated with the classical phenotype of Fabry disease. Intralysosomal deposits were found in all biopsies, corresponding to the skin, kidney, and endomyocardium in both sexes and at different ages. In nine of the skin biopsies, deposits were analysed by immunofluorescence and quantified at the ultrastructural level. Then, the findings were compared according to sex, genotype, and treatment. The quantification of the deposits in the skin biopsies revealed a broader involvement in men than in women. A significant clearance of the deposits was observed in one case after treatment. Tissue involvement was remarkable at diagnosis in all individuals. The findings from the skin biopsies were demonstrative of classic FD, thus supporting the diagnosis; repeated biopsy analyses suggested the benefit of early treatment.

## 1. Introduction

Fabry disease (FD) (OMIM#301500) is a rare metabolopathy with an estimated incidence of 1 affected male in 8454–117,000 [1]. It is transmitted by X-linked inheritance, and it is characterised by a deficiency in the lysosomal enzyme α-galactosidase A (AGAL, EC3.2.1.22) [2]. Consequently, incompletely degraded neutral glycosphingolipids, mainly globotriaosylceramide (Gb3), progressively accumulate in lysosomes in a wide variety of cell types [3]. Lysosomal and cellular dysfunction trigger a cascade of inflammation, microvascular injury, oxidative stress, tissue ischaemia, and cell death, thus leading to disease manifestations with predominantly cutaneous, cardiac, renal, and nervous system involvement [4].

Diagnosis of FD can be challenging, even within families, due to its high phenotypic variability. A demonstration of Gb3 deposits on target organs supports the diagnosis when enzymatic or genetic results are inconclusive. This procedure can shorten the time to diagnosis, which is estimated to be 15 years on average after the onset of symptoms. The length of diagnosis can lead to a delayed treatment and, therefore, a reduced therapeutic efficacy [5].

Approved therapies include enzyme replacement therapy (ERT) with recombinant AGAL (which has been used clinically since 2001 in Europe and since 2003 in the USA) and the pharmacological chaperone Migalastat, which has been available since 2016. However, questions remain about the ideal time to initiate treatment, the optimal dosage, and the treatment goals [6,7,8].

We report a histological and ultrastructural study of several biopsies from 11 individuals of a Spanish family who had been diagnosed, as confirmed by a genetic study, with classical FD. The objectives of this study were as follows: (a) to describe the findings of the biopsies of the different cell types examined under light and electron microscopy, as well as under immunofluorescence; (b) to quantify the lysosomal storage in fibroblasts and vascular endothelial cells in the skin biopsies; and (c) to assess the possible differences according to sex, genotype, phenotype, and treatment.

## 2. Materials and Methods

### 2.1. Patients

**Figure 1:**
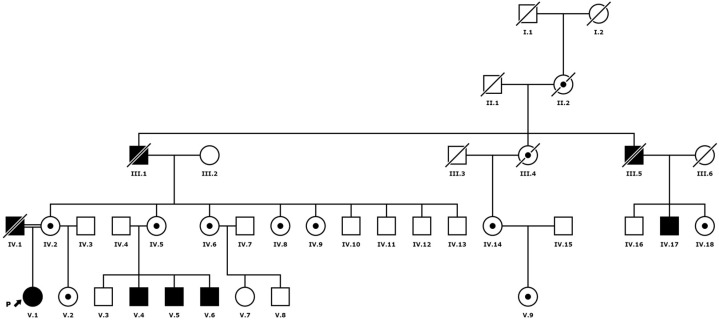
Family tree.

We studied a total of seven females and four males aged 20–47 years from two generations of a Galician family diagnosed with FD (Figure 1). Thirteen of the biopsies were examined by light and electron microscopy after informed consent was obtained. In 7 individuals (5 females and 2 males; Figure 1: IV.6, IV.14, IV.18, V.1, V.4, V.6 and V.9), 5 mm diameter punch biopsies of the unaffected skin from the lateral region of the trunk were performed under local anaesthesia. In two of them (Figure 1: V.1 and V.4), a second biopsy was obtained after 3 and 1 years of ERT, respectively. In 2 of the patients with left ventricular hypertrophy, i.e., a 50-year-old woman and a 46-year-old man (Figure 1: IV.5 and IV.17), endomyocardial biopsies were undertaken for diagnostic purposes. Two renal biopsies were studied in a 20-year-old woman before the start of treatment (Figure 1: V.2) and in a 40-year-old man who had been treated with ERT for 5 years due to severe renal failure and the development of inhibiting antibodies against recombinant AGAL (Figure 1: V.5) (this patient eventually required a kidney transplant).

### 2.2. Enzymatic and Molecular Study

Fabry disease was diagnosed on the basis of the clinical manifestations, or after a family study in search of possible new cases. The quantitative determination of the AGAL enzyme activity in the dried blood spots (DBS) was achieved using the Chamoles method [9]. Briefly, a fluorimetric assay was performed in triplicate on the DBS fragments, which had a 2 mm diameter, in a 0.15 M citrate-phosphate buffer at a pH of 4.2. This was performed while using 4-methylumbelliferyl-galactopiranoside (2.5 mM) as a substrate and when in the presence of N-acetylgalactosamine (0.25 M). The enzymatic activity was calculated via referring to a standard curve of 4-methylumbelliferon, and these were expressed as the μmol of the substrate per hour in a litre of blood (μmol/Lh) ± SD. A subsequent molecular analysis of the *GLA* gene was performed, whereby the exons of the GLA gene were amplified by PCR with specific primers, then sequenced via the Sanger method on an AB Prism 310 Genetic Analyzer (Thermo Scientific, Waltham, MA, USA). The results were analysed using Chromas 2.4 software, South Brisbane, Australia.

### 2.3. Histological and Ultrastructural Study

Half of each biopsy specimen was cut into small rectangular pieces, fixed in 2.5% glutaraldehyde in cacodylate buffer, washed carefully, postfixed in osmium tetraoxide, dehydrated progressively in graded acetones, and routinely embedded in Epon. In the case of skin biopsies, blocks were properly oriented to examine the entire depth of the dermis. Semithin sections were stained with toluidine blue and examined by light microscopy (LM). Ultrathin sections were mounted in copper grids, contrasted with uranyl acetate and lead citrate, and examined using a Talos L120C transmission electron microscope (TEM) Thermo Scientific, Waltham, MA, USA. A small fragment was fixed in 10% formaldehyde, embedded in paraffin, and stained with haematoxylin–eosin (HE) for LM examination. In some cases, small fragments of the skin biopsies were placed into a sterile culture medium for fibroblast culture and biochemical determinations.

The following cell types were examined: fibroblasts, endothelial cells, smooth muscle cells, pericytes, Schwann cells, perineural cells, epithelial and myoepithelial cells of sweat glands in skin biopsies, myocardiocytes in endomyocardial biopsies and podocytes, renal tubular epithelial cells, mesangial cells, and glomerular endothelium in kidney biopsies.

### 2.4. Direct Immunofluorescence with Gb3

A fragment of each biopsy was fixed in 4% paraformaldehyde and snap-frozen for an immunofluorescence study with Gb3/CD77 (clone 5B5, BD Pharmingen, Franklin Lakes, NJ, USA #551352). 40,6-diamidino-2-phenylindole (DAPI) and rhodamine-phalloidin were used to visualise the nuclei and membranes (polymerised actin), respectively. Immunofluorescence was performed on frozen sections 10 µm thick. The slides were permeabilised with phosphate-buffered saline (PBS), 5% bovine serum albumin (BSA) and 0.01% Triton. Images were captured using a conventional DM6 fluorescence microscope equipped with a DFC550 camera (Leica, Wetzler, DE, USA). A primary fibroblast culture was obtained by culturing a fragment of the skin biopsy in Dulbecco’s modified Eagle’s medium with the addition of 20% fetal bovine serum, 10% (*v*/*v*) penicillin, 1% (*v*/*v*) streptomycin, and 0.25μg/mL amphotericin B.

### 2.5. Storage Quantification in Skin Biopsies

Fibroblasts and endothelial cells were chosen for quantification of sphingolipid deposits at the ultrastructural level because they are easy to compare in different sections and cases. Twenty-five fibroblasts and 15 endothelial cells were imaged by TEM with an integrated Ceta CMOS digital camera (Thermo Scientific, Waltham, MA, USA), and the total cell area as well as the area of each lysosomal inclusion were measured in square microns (µm^2^) using NIS Elements Imaging Software 4.30.02 from NIKON, Tokyo, Japan.

The skin biopsy results were compared between females and males (7 and 4 cases, respectively), hemizygotes (Figure 1 IV.4 and IV.5), heterozygotes (Figure 1 III.5, III.6, III.14 and IV.9), and homozygotes (Figure 1: IV.1). The results were also compared between two women, one heterozygous (Figure 1: IV.9) and one homozygous (Figure 1: IV.1), whose biopsies were performed at the same age (24 years). Deposits were quantified in two patients whose biopsies were taken after 1 and 3 years of ERT with agalsidase beta (Figure 1: IV.1 and IV.4).

The Mann–Whitney test was used for statistical analysis of samples comparing two groups, and the Kruskal–Wallis test for analyses of three or more groups, with a significance level of *p* < 0.005.

## 3. Results

### 3.1. Patients

The age of patients at diagnosis ranged from 20 to 47 years, with a mean of 32 years in men and 34 years in women. The most common symptoms and signs are summarised in Table 1. Suspicion of FD was based on clinical manifestations or on the pedigree (Figure 1) of the index case (Figure 1: IV.1). The onset symptoms in males ranged from acroparesthesias, angiokeratoma, and episodes of neuropathic pain (“Fabry crisis”) in childhood and adolescence to symptoms of cardiac, renal, or psychiatric involvement in young adulthood. All male patients presented with coarse facial features, as did the homozygous female patients.

In general, manifestations in women were less severe, with abdominal pain, dyslipidaemia, and acroparesthesia being the most common, with the exception of a 21-year-old woman (Figure 1.IV.1) with angiokeratomas, hypohidrosis, and acroparesthesia since the age of 8, years, who had been examined from adolescence onwards for heart disease and cornea verticillata.

### 3.2. Enzymatic and Molecular Study

The determination of AGAL activity in DBS (Table 2) showed that the enzyme activity was reduced in the four males with the pathogenic variant in *GLA* (Figure 1: III.17, IV.4, IV.5, IV.6) and in one homozygous female (Figure 1: IV.1). All heterozygous females (Figure 1: III.2, III.5, III.6, III.14, III.18, IV.2, IV.9) had intermediate or normal levels of enzyme activity. The six healthy males of the family without the pathogenic variant had normal enzyme activity (Figure 1: III.10, III.11, III.12, III.13, III.16, IV.3, (Figure 1). 

Genetic testing was performed to confirm the diagnosis of FD in all subjects, and one woman was identified as homozygous for the missense variant NM_000169.3:c.836A > G in exon 6 of *GLA* (Figure 1:IV.1). This variant causes amino acid substitution p.Gln279Arg in the protein. Her father had died of chronic renal failure, and her 44-year-old mother (Figure 1:III.2) reported persistent paraesthesias and headaches.

**Figure 2:**
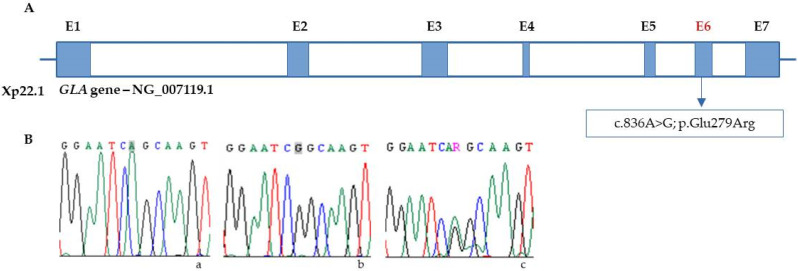
(**A**) Localization of c.836A > G variant in the *GLA* gene (E = exon). (**B**) Chromatograms from Sanger sequencing of *GLA* in (**a**) control; Adenine (A) highlighted in grey, green single peak (**b**) hemizygous male, Guanine (G), highlighted in grey, black single peak; (**c**) heterozygous female, Guanine/Adenine, (R), double peak green/black.

The pedigree (Figure 1) revealed inbreeding between her parents, and the genetic study confirmed the p.Gln279Arg variant in the mother and in four of her sisters (Figure 1: III.2, III.5, III.6, III.8, III.9), but not in any of her four male siblings (Figure 1: III.10, III.11, III.12, III.13). In the homozygous female generation, the pathogenic variant was detected in her half-sister (Figure 1: IV.2) and in three of her four male cousins aged 28, 29, and 30 years (Figure 1: IV.4, IV.5, IV.6) (Figure 2).

Almost a decade after the diagnosis of the index case, genetic testing confirmed the diagnosis in two women from another branch of the family, a 49-year-old woman (Figure 1: III.14) who was being investigated for proteinuria and heart disease and her 19-year-old daughter, who reported only occasional abdominal pain (Figure 1: IV.9). Neither was aware of a diagnosis of FD in the family, although the mother described a maternal history of heart disease.

Of the 14 individuals diagnosed by genetic study, ERT was initiated at a mean age of 41.2 years in females and 34.7 in males, with agalsidase alfa in six subjects and agalsidase beta in four. Two cases are currently awaiting treatment (Figure 1: III.6 and IV.9), and three refused treatment or medical follow-up after diagnosis (Figure 1: III.1, III.8, and III.9).

### 3.3. Histological and Ultrastructural Study

The main indication for skin biopsy was the presence of tissue involvement, especially in young or oligosymptomatic individuals. Cardiac and renal biopsies were performed in the presence of ventricular hypertrophy or renal failure of unknown origin. No changes in HE staining were observed in the skin with LM. Endomyocardial biopsies showed occasional clear perinuclear microvacuoles in myocardiocytes with LM, without inflammatory or fibrotic areas. Renal biopsies showed focal segmental glomerulosclerosis and chronic tubulo-interstitial nephritis.

**Figure 3:**
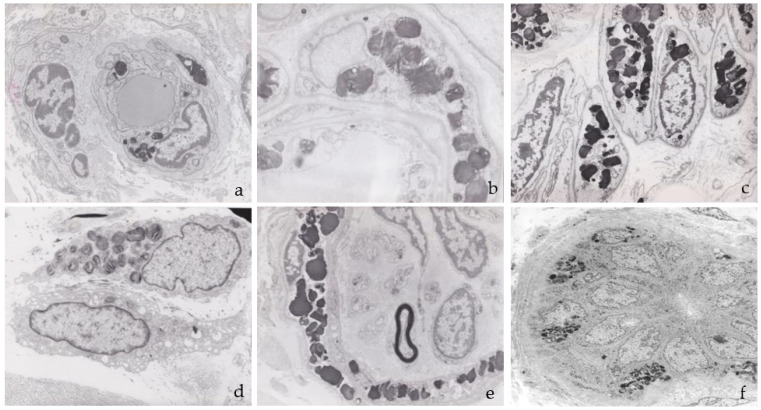
Ultrastructure of the deposits in skin biopsies: (**a**) capillary endothelium; (**b**) pericyte; (**c**) smooth muscle vascular cells; (**d**) fibroblast; (**e**) perineural cells of a myelinated nerve ending; (**f**) epithelial and myoepithelial cells of a sweat gland.

Ultrastructural examination of the skin samples showed electron-dense deposits in the endothelial cells, smooth muscle and pericytes of the vascular walls, interstitial fibroblasts, epithelial and myoepithelial cells of eccrine sweat glands, smooth muscle of the arrector pili, and perineural cells of myelinated and unmyelinated dermal nerve endings (Figure 3). No deposits were observed in axons or Schwann cells (Figure 3e). The deposits were membrane-bound due to their intralysosomal location and ranged in diameter from 0.6 to 1.3 µm in both sexes.

**Figure 4:**
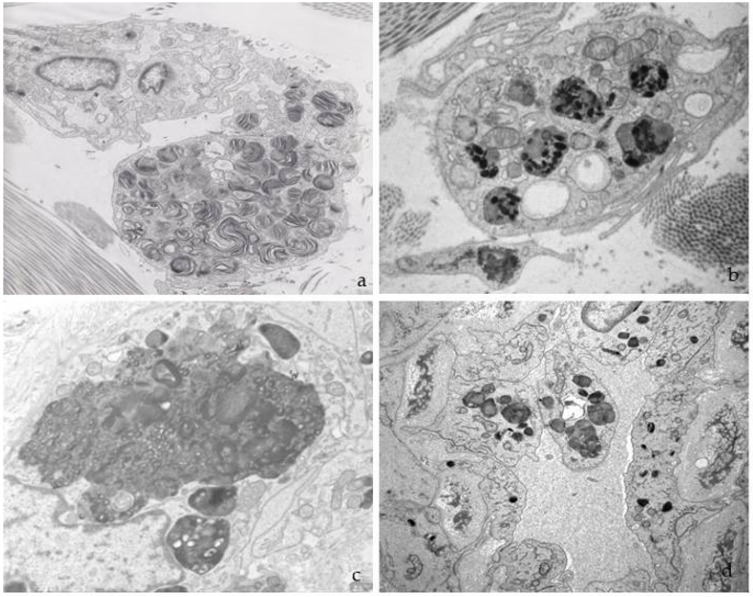
Variety of deposits under electron microscopy: (**a**) “Zebra bodies” in a dermal fibroblast; (**b**) lysosomes with inclusions of different morphology; (**c**) large deposit in an endothelial cell; (**d**) endothelial cell full of abnormal lysosomes, protruding into the vascular lumen.

The morphology of the deposits was variable, with the largest being electron-dense and compact (Figure 3 and Figure 4). Characteristic “zebra bodies” (Figure 4a), consisting of multilamellar structures with alternating electron-dense and electron-lucent parallel or concentric bands with a regular periodicity of 40–60 Å, were frequently observed. Deposits of heterogeneous morphology were sometimes present within a single lysosome (Figure 4b), whereas the lysosomal membrane was less prominent in larger deposits (Figure 4c). The increase in cytoplasmic volume caused by storage was more pronounced in the endothelial cells, which often protruded into the vessel lumens, reducing their calibre (Figure 4d).

**Figure 5:**
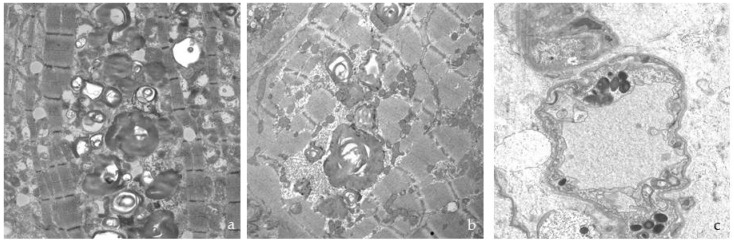
(**a**,**b**) Deposits found in an endomyocardial biopsy between myofibrils and (**c**) in the endothelium of a capillary.

In two myocardial biopsies, abundant electron-dense deposits were found in myocardiocytes, fibroblasts, endothelial cells, smooth muscle cells, and pericytes. In myocardiocytes, deposits formed large, electron-dense, and compact cytoplasmic masses that displaced myofibrils towards the periphery (Figure 5a,b). The loss of myofilaments was also observed. The deposits were often perinuclear and extended into the rest of the sarcoplasm, reaching large diameters and occupying a large portion of the cell surface.

**Figure 6:**
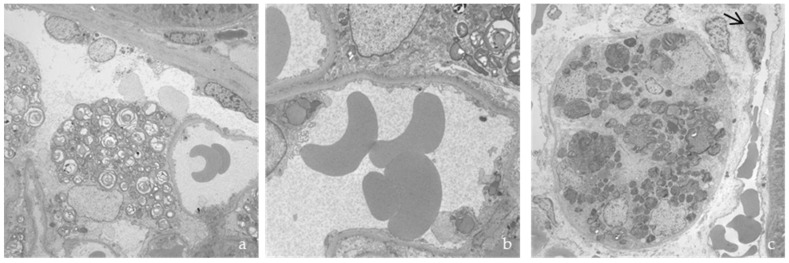
Renal biopsy, with deposits in (**a**) podocyte; (**b**) glomerular capillary; and (**c**) tubular epithelium and vascular endothelium (arrow).

The main ultrastructural alteration in renal biopsies was storage in the podocytes, mesangium, tubular epithelium, vascular walls, and fibroblasts (Figure 6), with effacement of the pedicels.

### 3.4. Direct Immunofluorescence with Gb3

**Figure 7:**
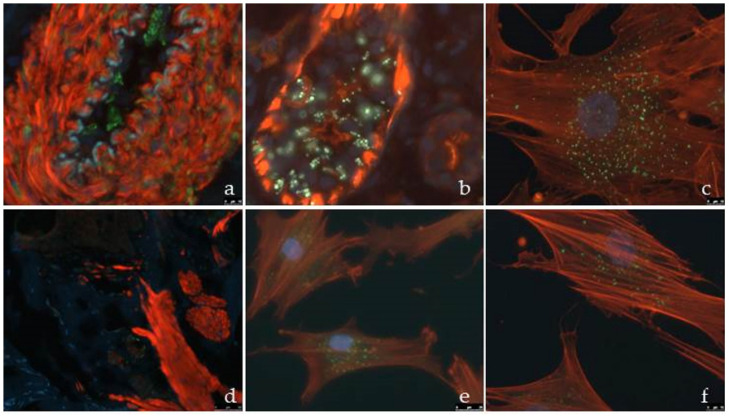
Gb3 immunofluorescence in (**a**) vascular endothelium; (**b**) sweat gland, and (**c**) cultured fibroblast from a skin biopsy of a symptomatic woman (Figure 1, IV.14). (**d**) Skin biopsy from her asymptomatic daughter (Figure 1, V.9), with no evident signal of Gb3 deposit. (**e**,**f**) Cultured fibroblasts from the skin biopsy of subject in (**d**) with presence of Gb3 signal. Elastic membrane: bluish green; Gb3 deposits: green.

Gb3 immunofluorescence of the skin biopsies demonstrated storage in the vascular endothelium and glandular epithelium (Figure 7a,b) in a symptomatic woman (Figure 1: III.14), as well as in a fibroblast culture (Figure 7c). In contrast, they were not visible by immunofluorescence (Figure 7d) in the biopsy of her asymptomatic heterozygous daughter (Figure 1: IV.9), but were prominent in the fibroblast culture (Figure 7e,f).

### 3.5. Storage Quantification in Skin Biopsies

**Figure 8:**
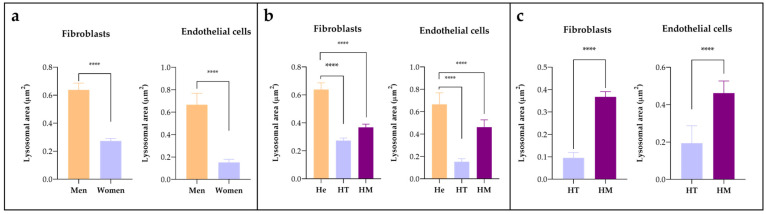
Comparison of the storage area in fibroblasts and endothelial cells between (**a**) men and women; (**b**) hemizygous (He), heterozygotes (HT), and homozygotes (HM); (**c**) comparison of heterozygote (HT) and homozygote (HM) women of the same age. **** *p* value < 0.0001.

Quantitative analysis of skin biopsies showed that the area occupied by deposits was significantly greater in males than in females (*p* < 0.0001), 57.2% greater in fibroblasts, and 77.1% larger in endothelial cells (Figure 8a). The biopsy with the smallest deposits was from a 19-year-old heterozygous woman (Figure 1:IV.9) who reported occasional abdominal pain and no organ involvement after her genetic diagnosis.

When the genotypes were compared (Figure 8b), the surface area of the deposits was larger in the homozygous woman than in the heterozygotes, although there were no significant differences found when comparing the homozygous woman (Figure 1:IV.1) with a heterozygous woman of the same age (24 years) (Figure 1:IV.9). This showed an increase of 73.9% in the fibroblasts and 58% in the endothelial cells (Figure 8c) in the homozygote.

**Figure 9:**
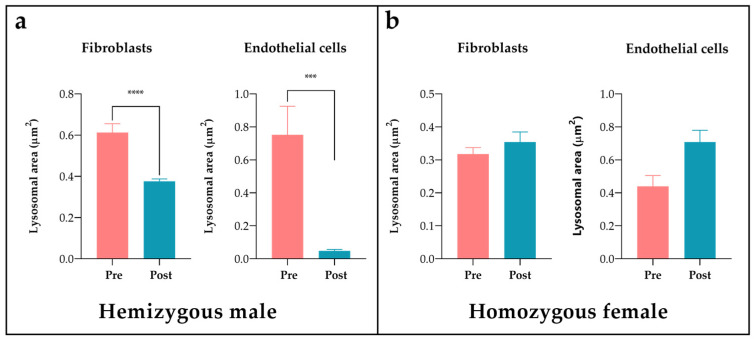
Storage clearance in fibroblast and endothelial cells after ERT in (**a**) a hemizygous male before (Pre) and one year after (Post) the start of treatment; (**b**) a homozygous female at the time of diagnosis (Pre) and after 3 years of treatment (Post). *** *p* value < 0.0005; **** *p* value < 0.0001.

Quantification of the deposits in the two patients with biopsies taken 1 and 3 years after ERT with agalsidase beta showed a significant reduction (*p* < 0.05) in the male patient after 1 year of treatment, with a clearance of 38.7% in fibroblasts and 93.6% in endothelial cells (Figure 9a). The patient had nephrotic-range proteinuria at the time of diagnosis at the age of 28 years; a renal biopsy showed focal and segmental glomerulosclerosis with interstitial fibrosis in addition to electrondense deposits at the ultrastructural level, leading to the diagnosis of FD. The patient was started on ERT two years later. Despite good compliance with the treatment and relative clearance of deposits in the skin biopsy, he developed high titres of antibodies to recombinant AGAL and the disease progressed to left ventricular hypertrophy and cerebellar stroke. It was decided to switch to agalsidase alfa, but this did not improve his tolerance to the recombinant enzyme. In view of the marked deterioration in renal function, a kidney transplant was performed in 2018, after which the patient progressed well.

On the contrary, no significant differences were observed in the skin biopsy of the homozygous woman after three years of treatment (Figure 9b).

## 4. Discussion

This study demonstrates extensive tissue involvement in skin, endomyocardial, and renal biopsies from 11 individuals of both sexes with different clinical manifestations who belonged to a family diagnosed with classical FD. The males presented with the classic phenotype, with underestimated signs and symptoms since childhood, such as neuropathic and abdominal pain, angiokeratomas, and hypohidrosis. Progression of the disease in adulthood, with signs of renal, cardiac, or central nervous system involvement, led to the diagnosis of the disease.

There was a significant delay from symptom onset to diagnosis in all cases, with an average delay of 13.25 years, resulting in late initiation of ERT. Early treatment has been demonstrated to reduce the pathological impact of the disease [6].

Screening for FD in at-risk populations with two or more clinical manifestations or a family history of the disease favours early diagnosis [10,11]. Significantly more abundant lysosomal deposits found in the skin biopsies of males in this study were correlated with lower plasma AGAL enzyme activity and a more severe clinical phenotype than those found in females, in agreement with other studies [12].

Phenotypic heterogeneity is a feature of FD, particularly in females, who present a wide spectrum from asymptomatic forms to severe phenotypes which are indistinguishable from those of males [13]. This can be explained by the somatic mosaicism status of females [14], according to the Lyon hypothesis [15,16] of random inactivation of one of the X chromosomes in each cell during embryogenesis.

The absence of symptoms in the youngest woman in this family, aged 19 years (Figure 1 V.9), and the presence of several oligosymptomatic women could be explained by instances of skewed X-chromosome inactivation (XCI) in favour of the non-mutated allele [17], although X-chromosome inactivation studies would be required to confirm these data. In other X-linked lysosomal storage diseases (LSD), such as mucopolysaccharidosis type II (Hunter’s disease), females are exceptionally affected, possibly because of the low penetrance in heterozygotes and cross-correction of the enzyme defect by cells carrying the active non-mutated allele [18,19]. In women with FD, the residual AGAL enzyme appears to be inadequate to correct the defect, either because the amount of the enzyme secreted is insufficient or because the affected cells do not take up the enzyme satisfactorily.

The female with the homozygous pathogenic variant, p.Gln279Arg, showed a severe phenotype and low enzyme activity, as is seen in hemizygous males. Upon quantitative analysis, the skin deposits were more abundant than those found in a heterozygous female of the same age (Figure 7c), although slightly less pronounced than that in the male group. Although the duration of treatment was too short to draw any conclusions, the lack of clearance of skin deposits after 3 years of ERT suggests advanced tissue involvement at the time of diagnosis, probably related to her homozygous status, without the attenuating effect of inactivation of one of the X chromosomes. Late initiation of treatment, as a consequence of delayed diagnosis at 24 years of age, as well as natural disease progression and the development of antibodies against recombinant AGAL may contribute to these results.

The quantification of clearance from cutaneous deposits in one man (Figure 1 IV.4) after 1 year of ERT was statistically significant in both endothelial cells and fibroblasts, particularly in the endothelium, in agreement with other studies [20], including the post-mortem examination of a patient treated for more than 2 years, in whom clearance was limited to endothelial deposits [21].

Despite significant clearance of Gb3 in the skin biopsy of this patient, his evolution was torpid, with deterioration of renal function and development of cardiac and CNS involvement, suggesting a limited effect of treatment, probably related to its late start at 30 years of age, when severe involvement was already present in the renal biopsy. In addition, the development of antibodies against recombinant AGAL may have contributed to its reduced efficacy [22,23].

Ideally, a larger number of cases with longer durations of treatment should be analysed in order to draw definitive conclusions. A phase 3 clinical trial with recombinant enzyme showed clearance of Gb3 from vascular endothelium after five months of ERT and a more gradual response in dermal smooth muscle and perineural muscle cells, correlating with the clearance of renal capillary deposits [24].

The efficacy of ERT has been shown to depend on the severity of organ damage, which in turn correlates with the time of treatment initiation [25,26,27]. Furthermore, impaired renal function at the start of treatment, with decreased glomerular filtration rate, proteinuria, and glomerulosclerosis, is considered an indicator of adverse development and the progression of renal involvement [28]. Other studies have shown that ERT is able to slow the progression of the disease, even in advanced stages [25].

Vascular dysfunction is a key pathophysiological mechanism in classical FD as a consequence of Gb3 deposition, with structural alteration of the vessel wall, endothelial activation, and induction of a prothrombotic state [29,30,31]. Progressive involvement of the microvasculature results in ischaemic damage, leading to renal and cardiac failure, as well as stroke [32,33]. It also reduces life expectancy by an average of 20 years in men and 15 years in women [3]. Given the systemic nature of vascular pathology, ultrastructural changes in skin biopsies are likely to reflect the degree of involvement in other tissues and organs.

The involvement of different skin structures explains some of the symptoms and signs that are often the first indications of FD, including angiokeratomas, acroparesthesia, or pain crises. In dermal capillaries, the progressive accumulation of Gb3 weakens the walls and leads to the formation of angiokeratomas, which are also seen in other lysosomal diseases such as fucosidosis type 2 [34], galactosialidosis [35], aspartylglucosaminuria [36], gangliosidosis 1 [37], β-mannosidosis [38], and Kanzaki disease [39]. Deposits in the epithelium of the eccrine sweat glands may be responsible for hypohidrosis, which is characteristic of FD [40,41].

In this study, the involvement of perineural, endothelial, and smooth muscle cells of the vascular walls, but not axons or Schwann cells, in patients with acroparesthesia and lancinating pain crises suggests an indirect effect of Gb3 deposits on the peripheral nerve via obstruction of the vasa nervorum, resulting in ischaemia [42]. Another alternative or contributing mechanism could be attributed to the effect of Gb3 deposition in sensory dorsal root ganglia [43,44].

Tissular deposits of Gb3 have been demonstrated in the podocytes, myocardium, cornea, placenta, and umbilical cord from very early asymptomatic stages of FD, even in the foetal stage [45,46,47,48,49], as well as in a skin biopsy from a 1-year-old child [50]. Deposition in the chorionic villi has also been described in other lysosomal storage diseases (LSD) such as Pompe disease [51]. Tissue lesions are already evident at the onset of symptoms, as demonstrated in the present study.

Although biochemical assessment is a well-established and inexpensive method for FD diagnosis and follow-up, the frequent detection of variants of unknown significance and the lack of correlation between genetic variants and clinical signs in patients identified through screening studies determine the need for confirmatory tests. As mentioned above, skin biopsies are accessible and non-invasive procedure that are useful for confirming diagnoses of FD and other rare diseases [52]. Ultrastructural examination of the skin has proven to be of great diagnostic value in other LSDs such as ceroidolipofuscinosis, some leukodystrophies, mucopolysaccharidoses, and even other complex diseases such as CADASIL [53,54]. Sample extraction can be easily performed by the physician and processed in the pathology department using standard fixation procedures. The sample can be shipped at room temperature to specialised diagnostic centres for ultrastructural analysis by TEM, or can be processed locally for Gb3 immunofluorescence. The total cost of this analysis is comparable to that of liquid chromatography–mass spectrometry (LC-MS) when used for the detection of Lyso-Gb3, which also requires sample shipment to specialised units.

During the traditional processing of paraffin-embedded tissues, sphingolipids dissolve, leading to cell vacuolization, which is more pronounced in cells that accumulate larger amounts of deposits, such as myocardiocytes or podocytes, but goes unnoticed in other cell types, such as those of the skin. In order to visualize the deposits and study their morphological characteristics, ultrastructural examination is required. Fixation in glutaraldehyde and embedding samples in resins preserves Gb3 deposits and allows for their morphological identification, as well as quantification of storage in different samples, which could eventually illustrate the effect of ERT [24].

Skin immunofluorescence showed Gb3 deposits only when they were abundant, whereas in fibroblast cultures, they were easily visualised even when they were scarce.

**Figure 10:**
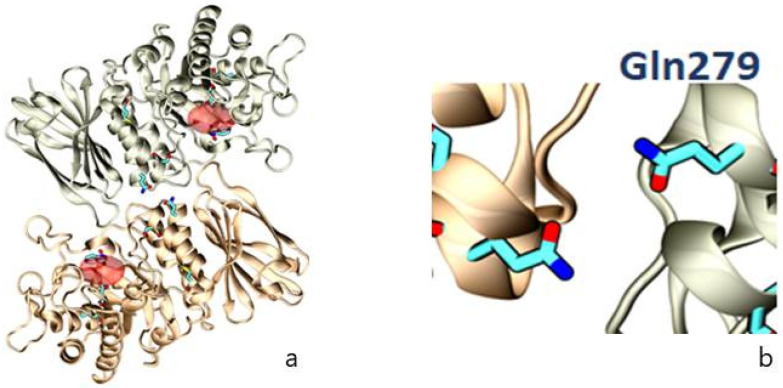
Representation of the crystallographic structure of AGAL as a homodimer, with (**a**) the position of selected amino acids (Leu131, Asp 170, Gln279, and Met290) involved in FD (in blue) and the active site (in red); (**b**) localization of the amino acid, which is affected in this family (Gln279), as a result of the missense variant NM_000169.3:c.836A > G.

Establishing a genotype-phenotype association in FD is complex due to the rarity of the disease and its allelic heterogeneity, with almost 1000 known *GLA* variants. The wide clinical spectrum of the disease and the lack of published data make this classification difficult. Our findings in this family with the p.Gln279Arg variant demonstrate its strong pathogenic character [55], which is likely related to the fact that the glutamine (Gln) residue is located close to the interface between the monomers, whereby the substitution of negatively charged Gln by positively charged arginine (Arg) would destabilise dimer formation (Figure 10). 

The current concept of the lysosome as a dynamic organelle with a much more complex function than catabolism alone, acting as an essential axis of cellular homeostasis [4,56], has revolutionised our understanding of LSD. We now understand that the deposition of undegraded material triggers a cascade of inflammation, oxidative stress, and altered immune responses, leading to irreversible cell damage, organ dysfunction, cell degeneration, and death, with a major impact on the development of a clinico-pathological phenotype [57].

Lysosomal dysfunction has a pivotal influence on human physiology beyond that of inherited diseases, being involved in the pathophysiology of common neurodegenerative and metabolic disorders, as well as cancer. It will most likely not be long before known diseases are classified as being of lysosomal origin [56].

The main limitation of the present study was the small sample size, an inevitable consequence of the rarity of FD, as well as the paucity of cases in which biopsies are performed. Differences in the stage of disease between patients and in the analysis of heterozygous, homozygous, and hemizygous data, or treated and untreated individuals, may have influenced the results. It would be interesting to correlate the ultrastructural findings with Gb3 or LysoGb3 levels in plasma or urine, as well as with AGAL activity at the time of biopsy, but these data were not regularly collected in the medical records.

In conclusion, this study demonstrates statistically significant differences in the quantity of dermal deposition between males and females with FD, which is consistent with the enzymatic levels of AGAL and the clinical differences between the two groups. We found that the behaviour of FD in a homozygous female is superimposable to that of a male with the classical form of the disease, and that the deposits are significantly more abundant than those found in heterozygous females, thus requiring early onset of ERT.

Despite advances in our understanding of the disease, the delay in diagnosis of FD remains considerable, and the introduction of new diagnostic techniques is imperative. Promising therapies which are currently in development show efficacy directly related to their early initiation, which would prevent the irreversible cellular damage that occurs in advanced FD.

Finally, the initiation of ERT before the onset of organ damage is essential for the clearance of Gb3 deposits, together with the development of strategies to reduce antibodies against the recombinant enzyme. This has been performed in cases of other LSDs, including Pompe disease [58]. It is, therefore, of great importance to study and screen as many individuals as possible once a case has been diagnosed in a family, not only to allow for early treatment, but also to facilitate appropriate genetic counselling to prevent further cases of this devastating disease.

## Figures and Tables

**Table 1:** Main symptoms in the group of men and women with Fabry disease. LVH: left ventricular hypertrophy.

	Men	Women
Angiokeratoma	3/4 (75%)	2/8 (25%)
Coarse facial features	4/4 (100%)	1/8 (12.5%)
Acroparesthesias	0/4 (0%)	3/8 (37.5%)
Pain crisis	2/4 (50%)	0/8 (0%)
Cornea verticillata	1/4 (25%)	1/8 (12.5%)
LVH	2/4 (50%)	2/8 (25%)
Proteinuria	3/4 (75%)	1/4 (25%)
Dyslipidaemia	1/4 (25%)	4/8 (50%)
Abdominal pain	1/4 (25%)	5/8 (62.5%)
Psychiatric symptoms	1/4 (25%)	4/8 (50%)
White matter abnormalities (MRI)	1/4 (25%)	1/8 (12.5%)
Lymphedema	3/4 (75%)	1/8 (12.5%)

**Table 2:** AGAL activity in DBS. F: female, M: male.

Case	Sex	AGAL Activity (µmol/L·h) *
IV.5	F	2.4 ± 0.12
IV.6	F	3.4 ± 0.32
IV.14	F	2.66 ± 0.48
IV.17	M	1.42 ± 0.07
IV.18	F	2.03± 0.54
V.1	F	1.82 ± 0.99
V.2	F	2.5 ± 0.42
V.4	M	0.75 ± 0.11
V.5	M	0.96 ± 0.05
V.6	M	0.87 ± 0.12
V.9	F	2.43 ± 0.27

* Reference value: 2.6–14 µmol/L·h, calculated in *n* = 15 healthy volunteers (11).

## Data Availability

Data are stored in the repository of Servizo Galego de Saude (regional public healthcare system), and are available upon request to the corresponding author.
